# Healthcare professionals' experiences of caring for women with false‐positive screening test results in the National Health Service Breast Screening Programme

**DOI:** 10.1111/hex.14023

**Published:** 2024-03-21

**Authors:** Hannah A. Long, Joanna M. Brooks, Anthony J. Maxwell, Sarah Peters, Michelle Harvie, David P. French

**Affiliations:** ^1^ Division of Psychology and Mental Health, Manchester Centre for Health Psychology University of Manchester Manchester UK; ^2^ Wythenshawe Hospital The Nightingale Centre, Manchester University NHS Foundation Trust Manchester UK; ^3^ Division of Informatics, Imaging and Data Sciences, School of Health Sciences University of Manchester Manchester UK

**Keywords:** breast cancer, breast screening, false‐positive screening test results, interview, mammography, qualitative

## Abstract

**Background:**

Understanding healthcare professionals' (HCPs) experiences of caring for women with false‐positive screening test results in the National Health Service Breast Screening Programme (NHSBSP) is important for reducing the impact of such results.

**Methods:**

Interviews were undertaken with 12 HCPs from a single NHSBSP unit, including advanced radiographer practitioners, breast radiographers, breast radiologists, clinical nurse specialists (CNSs), and a radiology healthcare assistant. Data were analysed thematically using Template Analysis.

**Results:**

Two themes were produced: (1) *Gauging and navigating women's anxiety* during screening assessment was an inevitable and necessary task for all participants. CNSs were perceived as particularly adept at this, while breast radiographers reported a lack of adequate formal training. (2) *Controlling the delivery of information to women* (including amount, type and timing of information). HCPs reported various communication strategies to facilitate women's information processing and retention during a distressing time.

**Conclusions:**

Women's anxiety could be reduced through dedicated CNS support, but this should not replace support from other HCPs. Breast radiographers may benefit from more training to emotionally support recalled women. While HCPs emphasised taking a patient‐centred communication approach, the use of other strategies (e.g., standardised scripts) and the constraints of the ‘one‐stop shop’ model pose challenges to such an approach.

**Patient and Public Contribution:**

During the study design, two Patient and Public Involvement members (women with false‐positive‐breast screening test results) were consulted to gain an understanding of patient perspectives and experiences of being recalled specifically in the NHSBSP. Their feedback informed the formulations of the research aim, objectives and the direction of the interview guide.

## 1 INTRODUCTION

Population‐based breast screening programmes aim to reduce breast cancer mortality by identifying cancers at an earlier and more treatable stage. In England, the majority of women will have a negative screening test result under the National Health Service Breast Screening Programme (NHSBSP), while a small percentage (approximately 4%) will have a screening test abnormality and be recalled for screening assessment for further investigations.^1^ These investigations can include clinical examination, repeat imaging (typically further mammography and ultrasound) and needle biopsy. Most recalled women (approximately 77%) will not be diagnosed with cancer and are thus said to have received a false‐positive screening test results (approximately 65,000 women annually in England).^1^

False‐positive screening test results are the most common harm experienced by women undergoing breast screening.^1^, ^2^ Systematic reviews of observational questionnaire studies have indicated that the receipt of false‐positive screening test results is associated with elevated breast cancer‐related anxiety and worry for women, which may be present up to 3 years later.^3^, ^4^, ^5^, ^6^, ^7^ A systematic review of the qualitative literature suggests that many women experience significant anxiety and worry about the need for additional tests (particularly biopsy) and the possibility of breast cancer and death.^8^ Receiving the ‘all clear’ typically brings significant relief for women, although some experience lingering, longer‐term worry.^8^ Reviews show mixed results regarding screening attendance after a previous false‐positive screening test result.^3^, ^5^, ^6^, ^8^, ^9^

It is unclear what role breast screening healthcare professionals (HCPs) might have in helping women manage negative emotions around the time of false‐positive screening test results. The qualitative literature indicates that women felt reassured by clear and thorough explanations of their false‐positive screening test results.^8^ However, some recalled unclear explanations from HCPs, perceived their result as a narrow escape from cancer, and reported lasting uncertainty.^8^, ^10^ There exists no specific guidance for HCPs for communicating results beyond ensuring that ‘assessment test results (whether normal, benign, or abnormal) are communicated clearly, accurately, and promptly, in person, by a member of the clinical team’.^11^ There has been little research into HCPs' experiences of caring for and communicating with women during assessment. HCPs are uniquely positioned to contribute novel insights into aspects of assessment, such as women's psychological responses to being recalled and the communication around results, as well as to the broader debate on the benefits and harms of screening.

The aim of this study was to understand screening HCPs' experiences of providing care for women during screening assessment, including their communication with women around false‐positive screening test results.

## 2 METHODS

### 2.1 Study design

This study used a qualitative cross‐sectional design.

### 2.2 Participants and setting

HCPs were recruited from a moderately sized NHSBSP screening unit in North West England. Of the c.431 small geographical areas served by this unit, 221 (51%) are areas of high deprivation.^12^ The original recruitment strategy was designed before the COVID‐19 pandemic and aimed to recruit up to 24 HCPs from two NHSBSP units (approximately 12 participants from each site) (see pre‐registered study protocol on the Open Science Framework doi.org/10.17605/OSF.IO/QWY2X).

HCPs were eligible if they (a) worked at the participating NHSBSP unit, (b) provided care for women during screening assessment and/or at biopsy results appointments and (c) were sufficiently fluent in English. Participants were recruited purposively to achieve diversity in professional roles. A range of HCPs was invited, including advanced radiographer practitioners (ARPs), breast radiographers, breast radiologists, clinical nurse specialists (CNSs) and radiology healthcare assistants.

### 2.3 Procedure

Two study collaborators at the unit emailed study invitations and information sheets to individual HCPs, and participated in interviews themselves. One reminder email was sent to nonresponding HCPs.

One‐to‐one interviews were chosen to facilitate an in‐depth exploration of experiences. Interviews were conducted by H. A. L. in private meeting rooms at the unit in January and February 2020. H. A. L. is a White British female PhD student of prebreast screening age, with 5 years of research experience and publications in the field of false‐positive‐breast screening test results.

An interview guide was developed based on relevant prior work^8^ and piloted with a consultant breast radiologist in the team (Supporting Information S1: 1). H. A. L. made field notes after each interview and kept a reflexive journal throughout the research. Participants provided written informed consent and completed a short demographic questionnaire (i.e., age range, gender) and gave details of their role (i.e., job title, years in role) before their interview. Interviews were audio‐recorded and transcribed verbatim. Participants were given pseudonyms and identifying information was removed.

### 2.4 Analysis

Data were analysed thematically using Template Analysis within a limited realist framework.^13^, ^14^ Template Analysis was selected for its ability to generate crosscutting themes in the data set, representing broad patterns in participants’ experiences with potential wider relevance.^13^, ^14^ H. A. L. led the analysis with input from the wider research team. Following *data familiarisation*, H. A. L. conducted *preliminary coding* on four transcripts. Codes were *organised into meaningful clusters* based on how they related to other codes within and between clusters. The *initial coding template was devised* and *applied iteratively to the remaining interview transcripts*. Further iterative coding and template revisions were completed as more data were analysed. A near‐final template was *applied to the full data set* again. The final template was agreed in team. See additional information in Supplementary Information S1: 2–4.

## 3 RESULTS

Twelve HCPs were recruited (Table 1). All were female. Interview duration ranged from 25 to 85 min (37 min mean).

**Table 1:** Sample demographic characteristics (*N* = 12).

Characteristic	*N* (%)
Age range (years)	
25–34	3 (25)
35–44	5 (42)
45–54	2 (17)
55–64	2 (17)
Healthcare professional role	
Advanced radiographer practitioner	2 (17)
Clinical nurse specialist	2 (17)
Consultant breast radiologist	3 (25)
Breast radiographer	2 (17)
Breast radiologist	1 (8)
Radiology healthcare assistant	1 (8)
Years in current professional role	
<1	2 (17)
1–4	7 (58)
5–9	1 (8)
20–24	2 (17)

Two themes were developed: ‘Gauging and navigating women's anxiety’ and ‘Controlling the delivery of information’.

### 3.1 Theme 1: Gauging and navigating women's anxiety

This theme summarises participants' experiences of assessing women's level of anxiety and navigating this during screening assessment. Most participants believed that women are generally unaware of and unprepared for the possibility of being recalled and the assessment process. This was reportedly because few women read and assimilated the breast screening leaflet and recall appointment letter.

A lot of the ladies that I've asked don't read the leaflet […] A lot don't know there's even a chance they could come back. (Serena, breast radiographer)

In participants' experiences, women attending follow‐up assessments are generally very anxious—a stark contrast to their relative ease at initial screening: “they're very anxious coming to this appointment” (Ada, CNS). At the beginning of the assessment, CNSs asked women to rate their anxiety level from 1 (no anxiety) to 5 (very anxious). Nancy, a CNS, shared:

I don't think we've ever had anyone score less than three […] most people are a five, because they are really anxious.

Participants believed that women perceive screening as a routine check for healthy women and mostly expect a normal screening result. However, when recalled, there is a significant shift in perception to ‘I (probably) have cancer’. Participants reported that women's anxiety is particularly elevated if they have a family history of breast cancer or are recalled after their first screening. Participants believed women are usually of the impression that recalls only happen when cancer is found or strongly suspected. Thus, women often interpreted the recall appointment letter to mean they have or are highly likely to have cancer: “they instantly think it's cancer” (Hattie, breast radiographer), “they always imagine the worst” (Ella, ARP), “some of them have booked their coffins” (Marie, ARP).

Participants acknowledged that the wait between receiving a recall appointment letter, attending assessment and, for some, receiving biopsy results is a time of high uncertainty for women, plagued with anxiety, worry and intrusive thoughts about the assessment process and the impact of having cancer.

But that waiting time plays more on you because you think, oh what are they going to do? What are they going to say? How are they going to do it? Will it hurt? (Ella, ARP)

Participants explained that efforts to provide reassurance can unravel when women are informed that a biopsy is required, with many women perceiving this as indicative of cancer, causing further anxiety.

I think sometimes it doesn't matter how much somebody says to you, ‘we don't think it's anything to be worried about but we want to do a biopsy’, the fact that they wanted to do a biopsy overrides what they've said before that. (Hattie, breast radiographer)

While participants agreed that all women experience some anxiety, they reported that the severity and expressions of anxiety varied considerably: “they're really, really, really varied and you don't know what they're going to be like until they arrive” (Marie, ARP). Mostly, participants relied on nonverbal communication to gauge women's distress level.

You can almost tell as soon as they stand up in the waiting room what sort of level they're at, because the body language normally says quite a lot. (Hattie, breast radiographer)

Understanding the extent of women's distress, easing their nerves and making them more comfortable during assessment was a high priority for all participants and necessary for performing further investigations adequately. Mae, a consultant radiologist, explained that biopsies can be technically challenging and this was compounded when women were anxious. Tensed, stiff muscles, heavy breathing, crying and fidgeting affected procedures by distorting image quality and moving biopsy targets.

At the end of the day my job is to get images that are adequate to help with the diagnostic process. That's my job but the woman is in the room, the woman is anxious, she is stressed, she is very nervous so for me personally that becomes my priority because there's no point, I can't get good images if she's nervous and shaking and that. So it's no point me even trying to do that until the patient herself is well enough. (Serena, breast radiographer)

The unit had recently implemented a 5‐min consultation with a CNS for all women at the beginning of assessment and an additional consultation afterwards for those undergoing biopsy. CNSs used this time to document women's medical history and anxiety scores, explain the reasons for the recall, prepare them for the assessment process, answer questions and provide reassurance: “it's to help alleviate and manage any anxiety” (Ada, CNS). CNSs extended these consultations to 10 min if additional support was needed.

All participants agreed on the positive impact of this initiative. Participants in other HCP roles perceived CNSs to be most skilled at alleviating distress, citing their formal training in this area. Women appeared less anxious after speaking with a CNS, and participants felt better prepared for consultations after reviewing CNSs' notes and women's anxiety scores. The radiologists collectively believed that this initial consultation with a CNS had improved the clinic's flow and allowed them more time to discuss the specifics of each woman's case. However, since the CNSs' had already covered medical history and addressed anxiety, they acknowledged fewer perceived opportunities during consultations to establish rapport and provide emotional support.

I think I used to detect more anxiety, so I used to have to spend more time in the non‐verbal and the verbal communication. But that's where the breaking of the ice and talking a bit about them and sometimes asking them about their jobs and their home circumstances, to reduce that anxiety level. I think that's been done by the breast care nurse now and I think it's great. We're not faffing around [laugh]. (Frida, consultant radiologist)

Participants described a need to work quickly. In their experience, the longer a procedure takes, the more distressed women become and the harder the procedure gets. Additionally, limited time is allocated per woman in the clinic. Participants described the time constraints of the ‘one‐stop shop’ approach to assessment clinics (whereby the aim was to complete all necessary tests on the same day). There is pressure to see women through all procedures as quickly as possible, while minimising additional distress.

The longer that you're pulling and pushing and twisting and they're in that uncomfortable position the less chance you've got of getting a good film. So you want to try and work as fast as you can because in terms of their anxiety levels it helps. (Hattie, breast radiographer)

The more ladies are waiting for us, the more anxious they're getting. So we have to streamline, we have to be efficient. You know, we can't spend 50 min with a patient when we've got 10 min. (Frida, consultant radiologist)

It's too much in a busy clinic, we can't spend too much time with everyone, trying to calm them down. (Sandy, radiologist)

Breast radiographers emphasised the importance of swiftly establishing trust with women to instill confidence in their competence at procedures, which helped to alleviate anxiety. However, a breast radiographer described feeling inadequately prepared by her training to provide the necessary emotional support to women, and had perceived this in newly‐qualified colleagues too. Other participants also acknowledged a relative disadvantage for radiographers. When asked how they developed the ability to give appropriate emotional support, several participants described a steep learning curve upon joining the screening service and a process of learning through trial and error over time and through experience in their role.

As a newly qualified, you're very focused, your images are going to be top quality, they're going to be the best images that you could do but you're not dealing with the emotions because you just can't. You haven't got the experience to deal with it. (Serena, breast radiographer)

### 3.2 Theme 2: Controlling the delivery of information

This theme describes how participants communicate with women during consultations. Participants perceived that the emotional strain of assessment often affects women's ability to absorb information effectively. Participants carefully managed the amount, type and timing of information provided to facilitate women's understanding and manage their anxiety.

Participants explained that women often feel overwhelmed during assessment. They are overloaded with information across several interactions with different HCPs during a time of distress. Their high emotions during the process and upon receiving their results were recognised as hindering their ability to fully engage in consultations and process information provided. Participants frequently recollected instances where they experienced women struggling to understand and remember information.

Usually there's a bit of ignorance about why we did the biopsy and sometimes, you know, people are telling you, but I think you are in such a state that you don't take in that information. […] everybody explained, just, you are just anxious, you didn't get that information. (Ella, ARP)

They only hear it's good news and they don't really recall anything else. (Greta, ARP)

With this in mind, participants described various communication strategies. Firstly, participants reported taking a ‘patient‐led’ approach in consultations with women. They gauged women's level of understanding and used this to determine how much information to share. They allowed themselves to be guided by women, disclosing only information they believed women wanted to know. These processes were active and explicit (e.g., directly asking women what they wish to know) and discreet and intuitive (e.g., reading nonverbal communication and interpreting questions asked indicative of interest).

I go by the patient, I don't try and tell them too much if they're not really bothered about being told. If they want to be told, I will go into it. (Marie, ARP)

Some patients don't want to know though, you get the feeling from them already that they're quite anxious and they're just very quiet and you just after a while of doing this, you just pick up that that patient doesn't want… [to know]. (Sue, radiology healthcare assistant)

Participants also described gradually drip‐feeding information to women. In their experiences, overloading women with potentially unnecessary, overly‐detailed information too early tends to hinder retention and increase anxiety. Participants deliberately conveyed only the necessary information at each stage, providing enough for women to understand the process.

So it's trying to give them enough information that they understand what's next what's happening but not too much that then gets their mind racing and thinking the worst. (Hattie, breast radiographer)

Participants described using standardised statements in consultations, seemingly developed through saying the same or similar information repeatedly. Sandy referred to her “routine spiel” and “standard phrases” (e.g., to explain the necessity of tests). Certain standardised empathic statements reflected an underlying attitude of prioritising caution and a ‘better safe than sorry’ approach to assessment, justifying the thoroughness exercised because a more advanced cancer in future is potentially prevented.

I'm really sorry that you've had to go through this again, but they want to make sure that they get the right result for you and that's important. (Sue, radiology healthcare assistant)

Participants described deliberate and careful word choices before results are known. They used open, vague and nonspecific language to explain what images indicated and the reasons for tests. They consciously avoided words perceived to be emotionally charged or loaded (e.g., ‘cancer’, ‘mass’). As only radiologists are qualified to interpret images and offer an opinion on results, other HCPs must use language that reflects the nature and limitations of their role.

We have to be quite vague when we first pull them in because we can't turn round and say so we've seen a mass in your right breast and it's about this size, because that's going to worry them a lot more. So we generally just say there's a small area within your right or left breast that the doctor wants to have a slightly closer look at and this is how I'm going to do it. (Hattie, breast radiographer)

Participants reported frequently repeating and reiterating information at each stage of assessment to aid retention. This was habitual and proactive—because, in participants' experiences, women are unlikely to remember without repetition—and responsive, when it was apparent women had not understood or taken in information well: “you can sort of tell how much they're taking in” (Rosa, consultant radiologist). While participants recognised the importance of ensuring women understood the process, not least for the purposes of taking verbal consent, some also recalled feeling frustrated at giving repeated information and answering repeated questions.

But then they will come to us, to our room, I will double explain to them what will happen and then I will tell them again why they have to have a biopsy. (Greta, ARP)

If I think that the patient is not really clicking, I'd tell the radiographers […] she's not really with it, so explain it again. Although I've explained everything, but explain it again. (Sandy, radiologist)

Given some women's difficulty retaining information, participants regularly invited women to ask questions to check their understanding and offer additional reassurance. Yet, in practice, participants sometimes needed to delay or avoid questions they were not permitted to answer. Participants also acknowledged the difficulty women have formulating questions while assessment is ongoing, instead believing that women would benefit from time to process their experience first. Participants encouraged women to telephone the CNSs with follow‐up questions, but both CNSs remarked that this service had been largely unused.

I get asked questions, ‘what's going to happen now?’ I get asked, ‘what do you think it is?’ I get asked, ‘so is it a mass? Is it small? Is it big? What happens if this does turn out to be cancer or something to worry about? Will they take my breast off? Can it be just taken out?’ All of those questions I can't answer, I have to get the doctor for. (Sue, radiology healthcare assistant)

A lot of them ask ‘there's a good chance this is benign?’ It's like prompting you to acknowledge that and I think you have to be careful there. (Frida, consultant radiologist)

Participants reported that radiologists made an early call on results when possible to alleviate some uncertainty and allow women to prepare for their results. They either delivered a ‘warning shot’ to women if a positive result seemed likely or cautious reassurance if a benign result seemed probable.

Sometimes you have an impression it's going to be benign, so you do tell them: okay, in my view, it looks like it's going to be benign but obviously we need to do a biopsy. (Mae, consultant radiologist)

The ARPs and radiologists described a defined process and standardised script for giving results. They aimed to share ‘good news’ promptly, bringing immense relief to women. From there, they gradually provided additional details, using simplified, lay language, and generally avoiding complex pathology details unless requested. They encouraged attendance at the next routine screening. Women are often elated to learn they do not have cancer and eager to leave. A few women ask questions, usually seeking clarification on the need for further tests, the certainty of results and whether they are at a greater risk of cancer and require more regular screening. Here, participants took the opportunity to reinforce the meaning of the result. Showing women their breast images helped to further evidence the benign finding.

More often than not, they are just so glad that you've said it's all okay, and some of them can't wait to get out of the room. […] There's not many that ask questions. The usual question is ‘will I have to come back more regularly now?’ (Marie, ARP)

## 4 DISCUSSION

Participants proposed that women experience anxiety because they generally perceive screening as routine, do not anticipate being recalled and fear that cancer will be (or has been) found. Participants emphasised the importance of patient‐centred communication, which appeared mainly limited to information giving (i.e. patient‐led information sharing, selectively‐dosing information), while also describing conflicting strategies (i.e. using standardised scripts). Participants perceived women's heightened emotions as interfering with information processing and retention. In participants’ experiences, women typically leave the clinic quickly and only a minority ask clarifying questions. Participants recognised the CNSs as particularly adept at building rapport and providing emotional support to alleviate anxiety. By contrast, breast radiographers reported a lack of adequate formal training on the emotional support of women, instead relying on time and experience to develop their skills.

The present findings are consistent with previous findings from systematic reviews of quantitative and qualitative studies of women with false‐positive‐breast screening test results, which indicate that women find it distressing to receive these results.^3^, ^4^, ^5^, ^6^, ^7^, ^8^ In line with previous research,^8^ women generally view screening as a routine service, do not routinely assimilate information supplied in advance and are unprepared for recall. Previous research shows that, within breast screening, women tend to overestimate the benefits and underestimate potential risks.^15^ While fully informed screening choices are a desirable goal, current NHSBSP practices to support this (e.g., via the information leaflet accompanying screening invitations)^16^ require women to have sufficient numeracy and literacy skills to process risk information and an understanding of their values and preferences. Moreover, these practices may not adequately account for the influences of emotion and affect decisions.^17^ Cancer fear is common amongst the pubic, perception of cancer risk often exceeds its actual level, and women's level of worry affects their subjective perceptions of breast cancer risk.^18^

The current findings raise questions regarding HCPs’ views on women's ability to fully accept the implications of a false‐positive screening test result, understanding these as an inherent screening limitation rather than cause for concern about their breast health or cancer risk. In line with previous findings,^19^ HCPs expressed the belief that women require time to process their experience before formulating follow‐up questions to address gaps in their understanding. While a potentially valuable service extension, the follow‐up telephone resource was largely unused at the present unit; thus, the nature of any residual concerns are unknown. Given some women return to routine screening with lingering concerns about their previous recall,^8^, ^10^ this also raises the question of the role of illness representations in women's understanding of and psychological adjustment to results.^20^, ^21^, ^22^ How do women make sense of their result and what if any lasting impact does this have on their cancer risk representations?

The present findings indicated that HCPs perceived the CNSs’ as particularly skilled at addressing women's emotional concerns. Recently, updated Public Health England guidelines^23^ on the clinical care of women recalled to assessment recommended a CNS assesses anxiety and offers appropriate support. Previous survey findings indicated that women given an opportunity to speak with a CNS before further investigations reported higher overall satisfaction with communication.^10^, ^24^ Further, women viewed quality communication with HCPs as the most stress‐relieving aspect of the assessment appointment.^10^, ^24^ It is plausible that future interventions to improve aspects of screening assessment for women could involve focusing on HCP communication practices.

HCPs in the present study were concerned with managing anxious women and the information they gave to women. Previous research indicates that the quality of communication and information provided is also important to women undergoing screening assessment,^8^, ^10^ as is the kind and supportive care of HCPs. Women with false‐positive screening test results have praised person‐focused care,^19^ describing nurturing HCPs as the best aspect of their screening experience,^25^ while others have acknowledged a lack of this as depersonalising.^26^ HCPs in the present study also described how the speed of appointments was important to minimise women's anxiety. While undergoing all necessary procedures on the same day is more practical, convenient, and cost‐effective, women's experience of the ‘one‐stop shop’ clinic model may be determined by the quality of care and communication they receive on the day.

The present findings offer novel insight. HCPs described strategies for communicating with women during assessment. Most patients’ preferred communication model in cancer care is patient‐centred.^27^, ^28^, ^29^ Patient‐led information sharing and gradual information‐dosing according to women's needs reflect elements of patient‐centred care,^30^ whereas the use of standardised scripts appears to be more HCP‐centred. In the present study, these relatively fixed scripts appeared to serve as ‘shortcuts’ for conveying key information and expressing empathy, potentially creating a tension between using scripts and maintaining genuinely patient‐centred communication. The present findings suggest that the ‘one‐stop shop’ model improves clinical efficiency, but its time constraints may limit a patient‐centred approach, potentially contributing to unmet needs for women.

## 5 STRENGTHS AND LIMITATIONS

The study protocol was preregistered (doi. org/10.17605/OSF. IO/QWY2X) and the report adheres to appropriate reporting standards (Supplementary Information 5).^31^ The interview guide was informed by relevant research^8^ and piloted before data collection. Analysis was conducted according to published methodological guidelines.^13^, ^14^

Participants were from a single NHSBSP unit. Eliciting the views of HCPs from other units would have been useful, as is not known whether the findings are representative of the wider NHSBSP. Originally, HCPs were to be recruited from another unit serving a larger regional catchment area.^32^ However, due to the impact of the COVID‐19 pandemic on NHS staffing capacity, this became unfeasible. Nonetheless, we recruited a wide range of HCPs (role, age, time in role), thus capturing diverse experiences of providing care at every stage of the assessment process.

## 6 PRACTICE AND RESEARCH IMPLICATIONS

Dedicated time with a CNS appears beneficial to women and valued by other HCPs. Units not yet following the relevant Public Health England guidelines^23^ may benefit from implementing this, if staffing capacity allows. Importantly, CNSs’ contributions may reduce perceived opportunities for other HCPs to build rapport with women. HCPs may view their emotional support as less necessary following support from a CNS. Explicitly providing emotional support before further investigations begin could mean emotional concerns arising during assessment are less likely to be addressed. It is important that emotional labour does not become solely the responsibility of CNSs (or any one HCP group), considering its association with emotional exhaustion, often a precursor to burnout.^33^

While involving CNSs in assessment is a positive step, it is crucial that all HCPs feel adequately equipped and supported to manage women's emotions. Participants expressed the view that breast radiographers possess the necessary technical imaging skills, but may lack skills and confidence in providing emotional support. Breast radiographers (especially newly‐qualified) may benefit from support and/or training in this,^34^ as communication skills do not reliably improve with experience alone.^35^ Given formal continuing professional development and training opportunities for radiographers are reportedly slim due to insufficient funding,^36^ it could be cost‐effective and pragmatic to include training at an earlier stage (e.g., prequalification) when there is motivation for learning and available training resources (e.g., simulated patient conversations, involvement of patients as educators).

Participants partly relied on a repertoire of standardised scripts to convey key information efficiently and effectively. Predetermined scripts may be useful for HCPs in difficult conversation (e.g., breaking the ‘bad news’ that a biopsy is necessary), but may inhibit tailored responses to informational (or emotional) needs in the moment. Participants' focus on information giving suggests this takes priority over other elements of patient‐centred communication. Participants acknowledged the challenges of providing emotional support alongside competing tasks in time‐pressured clinics, and thus scripts may serve a necessary purpose. Yet, there is research to suggest that addressing patients’ emotional concerns can save time in consultations.^37^ Future research could usefully investigate how HCPs develop scripts (e.g., through training, more intuitively, experientially, or by observing colleagues) and whether emotional concerns can be fully addressed without extending consultation times.

While the present findings offer novel insight into the nature of consultations during assessment, self‐reported communication practices may not ascertain the full or true nature of communication between HCPs and women. Future research using ethnographic observational methods and/or analysis of recorded consultations may permit the investigation of dyadic communication and offer insight into women's perspectives. Additionally, interviews with HCPs in other NHSBSP units may allow consideration of the representativeness of the present findings.

## 7 CONCLUSIONS

HCPs proposed that women experience distress when recalled to assessment because they were expecting a normal screening test result and fear that cancer has been found. Standardised scripts serve a necessary purpose in time‐limited consultations, but may not align well with patient‐centred communication. Support from CNSs' reportedly reduced women's anxiety and thus warrants a place in the ‘one‐stop shop’ assessment model. Breast radiographers may require support to develop skills to manage women's emotional concerns, addressing potential gaps in their formal training.

## AUTHOR CONTRIBUTIONS

**Hannah A. Long**: Conceptualisation; writing—original draft; project administration; formal analysis; methodology; data curation; investigation; writing—review and editing. **Joanna M. Brooks**: Conceptualisation; writing—review and editing; formal analysis; supervision; methodology. **Anthony J. Maxwell**: Conceptualisation; supervision; methodology; writing— review and editing; funding acquisition. **Sarah Peters**: Writing—review and editing. **Michelle Harvie**: Writing—review and editing; supervision; funding acquisition. **David P. French**: Writing—review and editing; supervision; conceptualisation; funding acquisition; formal analysis; methodology.

## CONFLICT OF INTEREST STATEMENT

The authors declare no conflict of interest.

## ETHICS STATEMENT

This study was reviewed and received a favourable opinion from the NHS South West Frenchay Research Ethics Committee (19/SW/0160) and the Breast Screening Programme Research Advisory Committee (BSPRAC 0082). Health Research Authority approval was received. All participants gave their informed consent to participate in the study and for the use of anonymised quotes in publications. To preserve anonymity, participants' names have been replaced with pseudonyms.

## Supporting information

Supporting information.

## Data Availability

The data set used in this study is not publicly available as it may contain information that would compromise participant consent. Please contact the corresponding author for more information. Further information on data analysis (including the procedure, reflexive practice, coding and every template version) is available from the Open Science Framework (doi:10.17605/OSF.IO/QWY2X).
