# Supplementary material for: Healthcare professionals' experiences of caring for women with false‐positive screening test results in the National Health Service Breast Screening Programme
Source: Health Expect. 2024 Mar 21;27(2):e14023. doi: 10.1111/hex.14023 (PMC10955228; doi:10.1111/hex.14023)
Supplement: Supplementary file 1 — Supporting information. [file HEX-27-e14023-s001.docx]

**Supplementary Information 1: Interview guide**

**
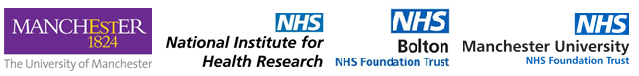
**

**Understanding women’s experiences of being recalled for more tests after an inconclusive screening in the NHS Breast Screening Programme**

**Interview schedule for healthcare professionals**

Key: **Key questions**- Probes
*Instructions to interviewer*

Introduction to interview:

- *Thank the participant for attending and introduce yourself.*
- *Explain that these interviews aim to understand more about women’s experiences of being recalled for more tests after an inconclusive screening result.*
- *Remind them that the interview will be audio recorded and transcribed.*
- *Remind them that names will not be used, but will be replaced with a false name, and any other identifying information (e.g. place names) will be deleted.*
- *Remind them that the interview could last approximately 30 minutes, they can stop at any time and do not have to answer every question.*
- *Remind them that if they share information about misconduct or poor practice, I have a professional obligation to report this and will need to inform their employer/professional body.*
- *Explain that there are no right or wrong answers; we are interested in what they have to say, both positive and negative comments.*
- *Provide the opportunity to ask questions.*
- *Check consent verbally and obtain written consent if the participant is happy to proceed.*
- *Give them a few minutes to complete the demographic questionnaire.*
- *Check that the recording device is working and turn it on.*

Part 1: experience of delivering false positive test results

**Could you tell me a bit about your role in the breast screening programme?**

**What involvement do you have with women when they are recalled for extra tests?**

- What are the priorities of your role when women come in for extra tests?
- What are you aiming for or trying to achieve?

**From your experience, how do you think women are feeling when they come in for extra tests?**

- How vocal are women about their feelings?
- What about at the biopsy results appointment? Any differences?

**What involvement do you have in explaining the results of extra tests to women?**

- What approach do you tend to take when explaining the results?
- What information or detail do you think is important to share?
- How much detail do you go into?
- How do you gauge what or how much information to share?
- Does this differ between results of tests performed at screening assessment and a biopsy results appointment?

**How do you prepare women who undergo a biopsy and have a period of time before the results of this?**

**Can you think of any changes or improvements to the Breast Screening Programme that could help or support women recalled for more tests?**

**Do women receive any information or advice about living a healthy life over the course of being recalled?**

- Do they receive any information or advice about reducing their chances of breast cancer?
- Do you think it would be appropriate to give women this information at this time?
- Who would be best placed to give it?
- What barriers might you encounter?

Part 2: views on the impact of being recalled on women’s future attendance

**From your experience, do you think the experience of being recalled affects women’s attendance at future screening?**

- Could you tell me a bit more about this?
- How do you think being recalled influence women’s feelings towards breast screening?

**From your experience, how do you think women feel when they return for their next routine screening, having previously been recalled?**

- Do they mention that they were previously recalled?
- Do they feel / behave / seem any different?

**Closing questions/comments:**

- Is there anything else you would like to share with me today?
- Do you have any questions for me?
- Remind them that if they have any questions after today they can contact you on the details provided in the information sheet.
- Remind them that if they would like a copy of the results after all the interviews have been completed they can let us know and you will send a copy of the final report.
- Thank them for taking part.

*Turn off the audio-recorder.*

**Supplementary Information 2: Final analysis template**

1. **Theme 1. Gauging and navigating women’s anxiety**
   1. **Women are anxious**
      1. **Women expect a normal result**
         1. Unaware of and unprepared for recall
         2. Do not read screening leaflet or recall letter properly
      2. **Recall letter interpreted as BC**
      3. **Mentally preparing for BC (death)**
      4. **A trigger for past experiences/events**
         1. Greater anxiety if family history of BC
      5. **Waiting time is very difficult**
         1. The ‘not knowing’
      6. **Difficulty reassuring women BC not certain**
      7. **Need for biopsy understood as sign of BC**
   2. **Every woman is different**
      1. **Spectrum of severity/expression of anxiety**
      2. **Gauging every woman**
         1. BCNS’ score anxiety
         2. Non-verbal cues indicate level of anxiety
         3. Silence indicates anxiety
         4. Lots of questions indicates anxiety
   3. **Dual role for professionals: juggling procedures and emotions**
      1. **Women’s wellbeing high priority**
         1. Importance of building rapport
         2. Putting women at ease
      2. **Competing clinical pressures and obligations**
         1. Technical challenges of anxiety
      3. **Other staff feel less qualified to manage anxiety**
         1. Emotional support and good communication perceived as nurses quality
         2. Value of time and experience in role
   4. **‘One stop shop’ care**
      1. **A well-oiled machine**
         1. Minimising waiting time
      2. **The key role of the BCNS**
         1. BCNS consultation(s): a service improvement
         2. Supports and prepares women
         3. Supports and prepares staff
2. **Theme 2. Controlling the delivery of information**
   1. **Women are overwhelmed at assessment**
      1. **Strong emotions prevents absorption of information**
      2. **Information overload**
   2. **Controlling the flow of information**
      1. **Gauging the conversation**
         1. Gauging level of understanding
         2. Gauging how much information to share
      2. **Staff drip-feed information**
         1. Anxiety management
      3. **Deploy pre-prepared, standard statements**
         1. Focus on procedural and practical information
         2. Better safe than sorry
      4. **Repeat information**
         1. Check women’s understanding (consent)
      5. **Use open, vague, simple language**
         1. Avoid loaded words
         2. Results not yet known
   3. **Difficulty processing and remembering information**
      1. **Invite and encourage questions**
         1. Staff expertise determines information sharing
         2. Women need time to process and reflect
      2. **BCNS telephone support: offered, encouraged, unused**
   4. **Communication around results**
      1. **Radiologists make early call on results**
         1. Fire warning/reassurance shots
      2. **Breaking the good news quickly**
         1. Standard method for delivering results
   5. **Closing the experience**
      1. **Reinforcing the benign result**
         1. Women keen to leave
         2. Questions need for tests and certainty of results
         3. Images used as (optional) evidence

**Supplementary Information 3**

**Additional information on data analysis procedure**

*Please find data analysis files referred to in-text in* **Bold** *on the study’s Open Science Framework page (doi.org/10.17605/OSF.IO/QWY2X)*

**Data analysis**

Data were analysed using a method of thematic analysis called Template Analysis (Brooks, McCluskey, Turley, & King, 2015; King & Brooks, 2017). A limited realist approach to inquiry was adopted, reflecting the position that “the world has a reality outside of human constructions of it, but that our understanding of it is always limited by our position within it.” (King & Brooks, 2017; pg. 18). We sought to understand what is true for participants, including their personal, subjective understanding and experiences, while also producing findings of value and wider utility for applied healthcare settings.

A defining feature of Template Analysis is the iterative development of a coding template (Brooks, McCluskey, Turley, & King, 2015; King & Brooks, 2017). King and Brooks (2017) offer a six-stage approach to Template Analysis, beginning with data familiarisation. In practice, I (HAL) read each interview transcript twice while listening to the audio-files. On my third read-through, I started actively engaging with the data, by – on printed transcripts – underlining excerpts, circling words, highlighting pauses and silences in participants’ dialogue, writing brief notes in the page margins and longer notes (e.g. any reflections, speculations, potential connections and underlying meanings) in a reflexive log.

As per King and Brooks’ (2017) procedure, preliminary coding is typically conducted on a subset of data. The idea is to capture a relatively wide range of participants’ experiences in the subset, as the codes assigned to these data are the foundation of the subsequent clustering and template structure (King & Brooks, 2017). The study team agreed that to achieve diverse experiences it would be pragmatic, to begin with, to select a participant transcript from each of the main healthcare professional groups sampled: breast radiographer, advanced radiographer practitioner, radiologist and clinical nurse specialist. Additionally, by this point I had a good understanding of the range of participants’ data and I thus selected further variation within the subset, based on the specific views and experiences participants’ shared.

Coding was inductive (data-driven) and involved highlighting short sections of data considered meaningful or relevant to the research aim and labelling these sections with descriptive and analytical codes (King & Brooks, 2017). This stage is a similar process as that used in most thematic approaches to coding qualitative data (King & Brooks, 2017). The first round of coding was performed on the printed transcripts, alongside noting further reflections and potential underlying meanings. Following this, a second round of coding was performed on blank (uncoded) Word document versions of the transcripts. This enabled me to revisit, repeat, compare and contrast against the codes assigned on the printed transcripts. I evolved existing codes and assigned new codes. This process was iterative; I moved continuously back and forth within and between the participants’ transcripts, creating and refining codes as I analysed more data.

The resulting codes were transferred to a Word document. The process had generated an enormous number of codes, with significant overlap and similarity (file **Preliminary codes 1**). To date, my experience in thematic analysis has been predominantly with a different style - Braun and Clarke’s. To organise codes, I was guided by Braun and Clarke’s (2013) guidance on reflexive thematic analysis that ‘good’ codes “capture the essence of what it is about that bit of data that interests you... and informative enough to capture what was in the data, and your analytic take on it” (pg 202). For example, the code ‘imagines the worst’ was deemed sufficiently captured by the code ‘women automatically assume/imagine breast cancer’ and thus deleted. Over a few revisions, the preliminary code list was clarified, organised and reduced in number (from 404 codes to 332 codes). To facilitate a study team analysis meeting with JMB, I loosely clustered and organised the codes based on similarity and in relation to the study aims (file **Preliminary codes 2**).

JMB (an experienced academic qualitative health psychologist and expert in Template Analysis) and I met to discuss the preliminary code list in greater depth. We began organising the codes into meaningful clusters based on how they related to other codes within (hierarchically) and between (laterally) clusters. We discussed tentative patterns in the data. The resulting document (file **Clustering**) is a relatively ‘big picture’ view, in that it indicates the early clusters of codes, but does not include all codes. Following this discussion, I further developed and formalised the clusters into a preliminary thematic arrangement and an initial coding template (file **Template 1**). Three themes and several sub-theme levels were created. An entry to my reflective log at the time indicates that this template reflects:

Me trying to play around and formalise what JMB and I discussed. My first proper go at putting codes into the right places, without doing too much editing. It shows how it would actually fit together and tell the story using the codes. Themes and sub-theme level names are new.

After further thought (going to and fro between the transcripts in the subset and the template; moving and renaming codes, sub-theme levels and themes) I devised a second template version (file **Template 2**). To facilitate a study team meeting with JMB and DPF, I drafted an accompanying narrative (written at the theme and sub-theme level), making additional changes to the template structure in the process. At this point, I created a longer version and a shorter version of the template (files **Template 3 shorter (2021)** and **Template 3 longer (2021)**). The former included every code under the relevant subtheme-levels, to retain detail and nuance, and the shorter ‘quick look’ version reflected the primary theme and sub-theme level structure and was intended to facilitate and support study team discussions. We discussed Template 3 in-team.

At this point, I took a period of leave from work and data analysis was suspended. Two years later, I resumed analysis. I sought advice from JMB regarding how to ensure integrity in the data analysis, given that it had started, stopped, re-started and was soon to be spliced back together. However, as JMB explained, an advantage of Template Analysis is the audit trail it leaves, clearly indicating the processes and decisions made. Following advice from JMB, I re-read all transcripts, code lists and template versions to facilitate data re-familiarisation. I closely reviewed the coded subset of transcripts, and re-coded these transcripts anew to record any areas of agreement and disagreement. I created approximately 40 new codes (file **New codes February 2023**) and discarded approximately 35 previous codes based on new and/or different interpretations of the data (evident in file **Template 3 longer_revised (2023)**). Otherwise, much stayed the same or changed only slightly. I had previously written a 4000-word narrative to accompany Template 3. I annotated this document with new ideas and interpretations.

In retrospect, Template 3 was an inadvertently useful stage to pause and resume analysis. This template version was detailed, thorough and descriptive yet relatively underdeveloped. The three themes appeared to be tentative, descriptive placeholders, rather than robust, analytical representations of the data. Structurally, it was somewhat flat and bottom-heavy with codes, lacking a compelling thematic hierarchy that suitably addressed the research aim. Consequently, between Template 3 and 4, a lot of work was done to build and refine the structure and to develop relatively distinct themes, while bearing the research aim in mind.

To do this, the revised Template 3 (incorporating the new codes) was used to iteratively code four further transcripts from a range of healthcare professional roles. New codes were created, existing codes were amended and similar codes were collapsed together to ensure the data was sufficiently coded. Template 4 was thus devised over a series of changes to codes, sub-theme levels and themes (files **Template 3.1**, **Template 3.2**, **Template 3.3**, **Template 3.4** and **Template 4**). It still contained three main themes and several sub-theme levels, but the ‘story’ of the analysis had developed. A supporting narrative with data excerpts was produced and this was discussed alongside Template 4 in-team with JMB and DPF. Two themes were generally agreed, but some sub-theme levels were moved, combined and clarified. The final theme was considered relatively unclear and lacking a coherent thread. Moreover, elements of this theme were considered potentially relevant to themes one and two. We agreed to amalgamate these parts into themes one and two where appropriate (file **Template 4 into Template 5**) and to ‘test’ the template on further new data.

Therefore, I made a temporary Template 5 and applied it to two further transcripts (file **Template 5 shorter_temporary**). Further coding was completed alongside structural changes to the template. The template itself was annotated with new ideas and connections (file **Template 5 longer_temporary_annotated**). JMB and I met to discuss the template in greater depth and made further amendments to the structure and names of codes, sub-theme levels and themes. We agreed that certain sub-theme levels needed greater delineation to capture the range of participants’ experiences and views. The order of sub-theme levels in theme two were moved around to change the narrative flow. Together, we applied Template 5 to the final transcript. One new sub-theme level was devised. Template 5 was finalised; the template and a revised accompanying narrative were discussed in-team with JMB and DPF (file **Template 5 shorter** and **Template 5 longer**). We agreed that Template 5 was a near-final template. Thus, as per King and Brooks (2017), Template 5 was applied to all transcripts again. Only minor changes were made and a near-near-final template was created (file **Template 6**). A revised narrative for Template 6 was produced. The template structure and study findings were agreed in-team with JMB and DPF.

Over the course of preparing the study manuscript for publication, parts of the template were ultimately removed from the Results narrative in order to meet journal article word count limit. Decisions regarding template amendments were discussed in-team and the manuscript underwent several revisions. Accordingly, this is reflected in the final and published template (file name **Template 7**). The final few revisions of the Results were agreed by all authors.

**References**

Braun, V., & Clarke, V. (eds). Successful Qualitative Research: A Practice Guide for Beginners. (Sage: London, UK, 2013).

Brooks J, McCluskey S, Turley E, King N. The utility of template analysis in qualitative psychology research. Qualitative research in psychology 12(2), 202-222 (2015).

King, N., & Brooks, J. (eds). Template Analysis for Business and Management Students. (Sage: London, UK, 2017).

**Supplementary Information 4**

**Additional information regarding reflexivity in data collection and data analysis**

**The researchers**

I (HAL) am a doctoral student in the field of health psychology. My doctoral research focuses on understanding the experience of receiving a false positive screening test result in the NHS Breast Screening Programme. Most of my qualitative research experience comes from formal degree programmes (i.e. BSc (Hons) Psychology, MSc Health Psychology and now PhD) and I gained some qualitative research experience as a Research Assistant prior to commencing my doctorate.

I have no experience of breast screening (I am outside the eligible age range). I have experience of cervical screening, including being recalled for further investigations. There is no history of breast cancer in my family. However, after data collection and before analysis, a close family member was diagnosed with terminal cancer and subsequently passed away. I was their carer during this time. This experience influenced parts of the study in obvious ways (e.g. affecting my feelings towards interviewing recalled women in the screening service about their experiences of ‘almost’ being diagnosed with breast cancer) and several less obvious ways (e.g. through my data interpretations and analysis decisions). I documented field notes after interviews and maintained a reflexive journal during data collection and analysis.

JMB is a senior lecturer in health psychology with expertise in qualitative methods and a number of publications on Template Analysis. She is co-supervisor of HAL’s PhD and has not participated in breast screening as she is outside the eligible age range. Her research interests include experiences of delivering healthcare and communication in healthcare settings, and she has previous experience of interviewing healthcare professionals (and subsequent data analysis) across a number of previous research projects. She has personal experience of caring at home for a close family member with terminal cancer and ongoing experience of living with a family member with long term health conditions which require ongoing healthcare service input.

DPF is a professor in health psychology with over 30 years of experience of conducting research into health services and psychological aspects of physical health. He is a supervisor of HAL’s PhD and is not eligible for breast screening due to his sex. He has conducted a substantial body of work on cancer screening, notably breast cancer screening, and is in receipt of numerous research grants relating to this service. He has personal experience of close family members having breast cancer diagnosed through screening.

We are broadly in favour of breast screening, but believe that the service can be improved to increase the benefits and reduce harms.

**Data collection (January to February 2020)**

I (HAL) made field notes immediately after interviews and reflective notes while listening to the interview audio-files before the next scheduled interview. These entries primarily focused on my data interpretations, questions that arose, notable points or ideas that warranted further thought or exploration in subsequent interviews, the dynamic between the participants and myself, and my interviewing technique. For example, after a few interviews, I perceived challenges in maintaining my neutrality and impartiality, fearing I might inadvertently reveal my personal opinions or beliefs about the impact of screening to participants and influence their responses during interviews. I wondered about adapting myself in interviews, and whether I could still adequately build rapport, encourage (but not endorse) participants using greater nonverbal communication (e.g. nodding, smiling) and active-listening skills (e.g. maintaining eye contact, asking thoughtful follow-up questions). My reflective log at this time indicates:

Be careful not to share my opinion too much – it is okay to paraphrase participants to check my understanding, but not to put words in their mouth or express my opinions or feelings. Don’t agree too strongly - “same” or “absolutely” is potentially inappropriate. Also, don’t respond “yep” in a way that makes it sound as though you already know their experience, what they are going to say and what they are talking about – it might shut them down. Nod instead to encourage them to keep going.

On reflection, I realised an internal conflict. I was aiming to minimise or erase my influence on the interview process and the data collected, yet my neutrality was performed, rather than natural, and I perceived it as a barrier to building rapport. Achieving both felt unlikely. I naturally rely on expressive positive verbal and nonverbal communication to build relationships in social interactions, but I felt the need to restrain these tendencies to be a ‘good’ interviewer. Consequently, I was struggling to find the right approach for rapidly building rapport (in the few minutes before and during an interview), encouraging participant engagement, while maintaining a ‘professional’ interviewer stance – which I then understood as relatively detached.

I sought advice from JMB, who clarified that maintaining total impartiality was not always necessary and that building rapport sometimes involves sharing personal experiences and beliefs. This gave me ‘permission’ to be more authentic and reminded me that complete removal of oneself in an interview is not possible or necessarily beneficial. I started being more open with participants from then on (e.g. about myself and my opinions on my PhD research thus far). Overall, I felt the interviews went well.

Field and reflective notes enabled me to document and examine my thoughts, responses and assumptions. This critical introspective and self-reflective process helped me to identify alternative ways of framing questions, responses and probes. It enabled me to consider my contribution to the research process and the construction of data.

**Data analysis (February to March 2021 and February to June 2023)**

Data analysis was conducted in two stages during a challenging time in my life. Prior to each stage, I felt anxious about reengaging with the data, given the crossover between recent personal events related to cancer and my research in cancer screening, as well as the immersive, intense nature of qualitative data analysis. Upon reflection, while the emotional toll was not as significant as anticipated, I noticed new thought processes and emotional responses to the data that required my ongoing awareness throughout analysis.

Specifically, between data collection and analysis, I experienced the stress of diagnostic uncertainty. Waiting for potentially life-changing appointments, consultations, tests and results for someone you love can be extremely difficult. My reflective notes indicate a shift in my perspective towards women recalled in screening. I began to empathise with and relate to them in a new way, feeling ‘on their side’ and developing an ‘us vs. them' attitude that was previously absent and contrary to the strong bond I felt with participants during data collection. During data analysis, I sometimes felt difficult negative emotions (e.g. moments of frustration or anger) in response to participants’ experiences and feelings, projecting my offence onto the participants. For example, some participants expressed surprise and bewilderment at the number of women who immediately ‘assume the worst’ and believe they have breast cancer upon notification of a recall appointment. When sharing their difficulty reassuring women that cancer is not a given, some participants gave the impression that some women cannot be reassured or reasoned with and are therefore at fault. I found it hard to relate to their surprise. I questioned whether participants’ comments implied they perceived recalled women as overemotional and irrational because they are experiencing something akin to a cancer diagnosis before their results are known. I questioned whether women’s feelings are thus invalidated because they are perceived as failing to approach the situation rationally and logically. Additionally, I was surprised that some participants expressed frustration at having to repeat themselves to distressed women who were struggling to absorb information given during screening assessment. These patterns in the dataset provoked feelings of misplaced anger within me. I wanted to see endless empathy and patience from healthcare professionals for women who, for various reasons, may find it easier or safer to assume the worst in the face of uncertainty and loss of control, and who were so stressed that they were unable to retain information as usual. Ultimately, I was processing my own experiences and feelings as, shortly before, I had felt similarly to the women that participants’ were recollecting.

These feelings surfaced during both stages of data analysis. During these moments, I needed to pause, actively remind myself that participants’ experiences and feelings were equally valid and, from a researcher perspective, these findings were interesting and novel. It is important to note that very few people possess unlimited empathy and patience; I had to be careful not to ‘over analyse’ these sections of data or assign more significance than warranted. This experience was more intense during coding, when I was particularly immersed in the data, but its impact lessened over time. The study team thoroughly reviewed the template structure and accompanying narrative multiple times, providing many opportunities to explore the rationale behind my analytical decisions and inferences.

**Supplementary Information 5 – COREQ statement**

|  | **Item No.** | **Guide Questions/Description** | **Reported on**  **Page No.** |
| --- | --- | --- | --- |
| **Domain 1: Research team**  **and reﬂexivity** | | | |
| *Personal characteristics* | | | |
| Interviewer/facilitator | 1 | Which author/s conducted the interview or focus group? | 3, Supp Mat 4 |
| Credentials | 2 | What were the researcher’s credentials? E.g. PhD, MD | 3, Supp Mat 4 |
| Occupation | 3 | What was their occupation at the time of the study? | 3, Supp Mat 4 |
| Gender | 4 | Was the researcher male or female? | 3, Supp Mat 4 |
| Experience and training | 5 | What experience or training did the researcher have? | 3, Supp Mat 4 |
| *Relationship with*  *participants* | | | |
| Relationship established | 6 | Was a relationship established prior to study commencement? | Supp Mat 4 |
| Participant knowledge of  the interviewer | 7 | What did the participants know about the researcher? e.g. personal  goals, reasons for doing the research |  |
|  |  |  | Supp Mat 4 |
|  |  |  |  |
| Interviewer characteristics | 8 | What characteristics were reported about the inter viewer/facilitator?  e.g. Bias, assumptions, reasons and interests in the research topic |  |
|  |  |  | Supp Mat 4 |
|  |  |  |  |
| **Domain 2: Study design** | | | |
| *Theoretical framework* | | | |
| Methodological orientation and Theory | 9 | What methodological orientation was stated to underpin the study? e.g. grounded theory, discourse analysis, ethnography, phenomenology, content analysis |  |
|  |  |  | 4 |
|  |  |  |  |
| *Participant selection* | | | |
| Sampling | 10 | How were participants selected? e.g. purposive, convenience,  consecutive, snowball |  |
|  |  |  | 3 |
|  |  |  |  |
| Method of approach | 11 | How were participants approached? e.g. face-to-face, telephone, mail, email |  |
|  |  |  | 3 |
|  |  |  |  |
| Sample size | 12 | How many participants were in the study? | 1,4, Table 1 |
| Non-participation | 13 | How many people refused to participate or dropped out? Reasons? | No data |
| *Setting* | | | |
| Setting of data collection | 14 | Where was the data collected? e.g. home, clinic, workplace | 3 |
| Presence of non-  participants | 15 | Was anyone else present besides the participants and researchers? |  |
|  |  |  | 3 |
|  |  |  |  |
| Description of sample | 16 | What are the important characteristics of the sample? e.g. demographic  data, date |  |
|  |  |  | Table 1 |
|  |  |  |  |
| *Data collection* | | | |
| Interview guide | 17 | Were questions, prompts, guides provided by the authors? Was it pilot tested? | 3, Supp Mat 1 |
|  |  |  |  |
| Repeat interviews | 18 | Were repeat inter views carried out? If yes, how many? | n/a |
| Audio/visual recording | 19 | Did the research use audio or visual recording to collect the data? | 3 |
| Field notes | 20 | Were ﬁeld notes made during and/or after the interview or focus group? | 3, Supp Mat 4 |
| Duration | 21 | What was the duration of the inter views or focus group? | 4 |
| Data saturation | 22 | Was data saturation discussed? | n/a |
| Transcripts returned | 23 | Were transcripts returned to participants for comment and/or | n/a |
